# *C. elegans* orphan nuclear receptor NHR-42 represses innate immunity and promotes lipid loss downstream of HLH-30/TFEB

**DOI:** 10.3389/fimmu.2023.1094145

**Published:** 2023-02-13

**Authors:** Debanjan Goswamy, Xavier Gonzalez, Sid A. Labed, Javier E. Irazoqui

**Affiliations:** Department of Microbiology and Physiological Systems, UMass Chan Medical School, Worcester, MA, United States

**Keywords:** *Caenorhabditis elegans*, *Staphylococcus aureus*, nuclear receptor (NR), TFEB, host response, infection, innate immunity, lipid droplets (LD)

## Abstract

In recent years, transcription factors of the Microphthalmia-TFE (MiT) family, including TFEB and TFE3 in mammals and HLH-30 in *Caenorhabditis elegans*, have emerged as important regulators of innate immunity and inflammation in invertebrates and vertebrates. Despite great strides in knowledge, the mechanisms that mediate downstream actions of MiT transcription factors in the context of innate host defense remain poorly understood. Here, we report that HLH-30, which promotes lipid droplet mobilization and host defense, induces the expression of orphan nuclear receptor NHR-42 during infection with *Staphylococcus aureus*. Remarkably, NHR-42 loss of function promoted host infection resistance, genetically defining NHR-42 as an HLH-30-controlled negative regulator of innate immunity. During infection, NHR-42 was required for lipid droplet loss, suggesting that it is an important effector of HLH-30 in lipid immunometabolism. Moreover, transcriptional profiling of *nhr-42* mutants revealed wholesale activation of an antimicrobial signature, of which *abf-2, cnc-2*, and *lec-11* were important for the enhanced survival of infection of *nhr-42* mutants. These results advance our knowledge of the mechanisms by which MiT transcription factors promote host defense, and by analogy suggest that TFEB and TFE3 may similarly promote host defense *via* NHR-42-homologous nuclear receptors in mammals.

## 1 Introduction

Recent years have witnessed the emergence of MiT transcription factors as key regulators of innate immunity genes during a wide range of disease conditions, including infections by bacteria and viruses and chronic inflammation (1). Mostly thought of as master regulators of lysosomal biogenesis and autophagy, their functions include positive regulation of lipid mobilization and cytokine induction (2–9). While overall the genes that MiT factors control in different scenarios are becoming better understood, their mechanisms of regulation and action are much less so.

To address these knowledge gaps, we apply the model organism *C. elegans* in the context of intestinal infection by *S. aureus* and other human and nematode pathogens (8, 10). In *C. elegans, S. aureus* infects the intestinal lumen by the natural oral route and causes a 7-fold reduction in host lifespan, which is the result of a pathogenic process that involves enterocyte effacement, intestinal epithelial destruction, and organ consumption within the span of 48 h (10). In previous studies, we identified two main genetic pathways that promote the induction of an infection-specific gene expression signature that promotes host survival (11): the acetylcholine-Wnt pathway (12, 13) and the TFEB/HLH-30 pathway (8, 14). Like its human and mouse counterparts, HLH-30 resides in the cytoplasm in homeostasis and accumulates in the nucleus of cells throughout the animal during *S. aureus* infection (8) and other stresses (4, 5, 15–19). We also understand that HLH-30 activation by infection requires the activity of an upstream genetic pathway that involves EGL-30/Gαq and DKF-1/Protein kinase D, which is conserved in murine macrophages (14).

Although 95% of the infection-specific signature is HLH-30 dependent (11), a vast majority of its genes lack HLH-30 cognate motifs in their regulatory sequences (8, 20). This raises the possibility that HLH-30 may exert its pro-host defense action through intermediate regulators of gene expression, likely transcription factors themselves. Indeed, we showed that HLH-30 induces the expression of 17 transcription factors during *S. aureus* infection (8), of which the majority are orphan nuclear hormone receptors (NHR).

The *C. elegans* genome encodes an expanded family of 284 *nhr* genes, compared to 48 in humans and 49 in mice, but only around 20 of these genes have assigned functions (21). In invertebrates and vertebrates, nuclear receptors can induce or repress target gene transcription depending on their structure, protein-protein interactions, and ligand binding (21). In *C. elegans*, specific nuclear receptors have been showed to regulate metabolism, reproduction, lifespan, and development (22). Recent studies have begun to uncover important roles for NHR in *C. elegans* host defense against infection (11, 23–29).

Here we report the unexpected finding that infection-induced HLH-30-dependent gene *nhr-42* is an important negative regulator of host infection resistance, which functions at the site of infection, *i.e.* the intestinal epithelium. During infection, we find that NHR-42 is essential for lipid droplet loss, suggesting that rather than through direct induction of lipid mobilizing genes, HLH-30 functions through NHR-42. In noninfected animals, NHR-42 represses specific genes that encode antimicrobial peptides and limits host survival in infected animals. These observations support the view that HLH-30 (and, by analogy, possibly TFEB and TFE3) induces transcription factors that are capable of repressing the host response and promoting the mobilization of lipids from intestinal lipid stores, with important implications for the resolution of infectious inflammation and for immunometabolism.

## 2 Materials and methods

### 2.1 *C. elegans* strains and growth

Strains used in this study are detailed in **Table 1**. C*. elegans* was grown on nematode-growth media (NGM) plates seeded with *E. coli* OP50 at 20°C, according to standard procedures (30), unless mentioned otherwise.

**Table 1:** List of C. elegans strains and oligonucleotides.

*C. elegans* (relevant genotype)	Source	Identifier
Wild type, Bristol isolate	*Caenorhabditis* Genetic Center (CGC)	N2
*nhr-42*(tm1375) V	National BioResource Project (NBRP), Japan	tm1375
*hlh-30*(tm1976) IV	Irazoqui laboratory	JIN1375
jinEx2291[P*nhr-42::mCherry*]	This study	JIN2291
*hlh-30*(tm1976) IV*; nhr-42*(tm1375) V	This study	JIN2292
*sid-1*(qt9) V; alxIs9 [P*vha-6::sid-1::SL2::GFP*]	CGC	MGH171
*rde-1*(ne219) V; kzIs9 [(pKK1260) P*lin-26::NLS::GFP* + (pKK1253) P*lin-26::rde-1* + *rol-6*(su1006)]	CGC	NR222
*rde-1*(ne219) V; kzIs20 [P*hlh-1::rde-1* + P*sur-5::NLS::GFP*]	CGC	NR350
RT-qPCR Oligonucleotides		
*nhr-42* F- GGGTCGCCGGATGCATATG R- GCAACATTGGGAGACGTGTGTTTC	IDT	
*hlh-30* F-GAACACATCAGAAGACATGAAAC R- AAGATGCGATGGCGGGACCT	IDT	
*abf-2* F- GGCTCAGGGGTTGTGCATTA R- GACGACCGCTTCGTTTCTTG	IDT	
*cnc-2* F-ATGATGGGAGGTTATGGAGGA R- CAAGGAGTCCAGGGCGATAC	IDT	
*irg-5* F- CGTACTCCATCCGATTCGCT R- GGTCGTACTTCTTCACCGCA	IDT	
*pals-23* F- AAGCTAGAAGGAGCACGACG R-GTGTTGTTGACAATGTGACGTG	IDT	
*cnc-4* F- GCTTCGCTACATTCTCGTCC R- TATGGACCGTAGCCCCATTG	IDT	
*lec-11* F- ATGCAAATGCCTGTTGCTCC R- CCGGAACAATCTGTGGTTGG	IDT	
*fmo-2* F-ATAATGAACACGCGTTTCTTC R-GATGTTTGGCTTGATTCTGA	IDT	
*snb-1* F-GAATCATGAAGGTGAACGTGG R-GAATGACGACGATAGCGCAC	IDT	
PCR Oligonucleotides		
P*nhr-42:mcherry* F- GAGCAGAGTTCGAGAATGTGC R-GTCAATGCAAAGTTGACACCGGG	IDT	

### 2.2 *C. elegans* bacterial infection assays

#### 2.2.1 Staphylococcus aureus infection

A single colony of SH1000 strain was grown in tryptic soy broth (TSB) supplemented with 50 µg/ml kanamycin overnight at 37°C shaking at 200-220 RPM. 10 µl of the overnight culture was spread on tryptic soy agar (TSA plates) supplemented with 10 ug/ml kanamycin. These plates were grown at 37°C for 5 h and then shifted to 25°C overnight*. C. elegans* were treated on solid media with 100 μg/ml 5-fluoro-2′-deoxyuridine (FUDR) at L4 larval stage overnight at 15°C before transfer to *S. aureus* TSA plates with full lawns of bacteria. Three TSA plates each containing 30-40 animals were examined for each genotype of *C. elegans*. Infection assays were carried out at 25°C as described (30). For RNAi experiments animals were grown to L4 larval stage on *E. coli* strain HT115 expressing double-stranded RNA for the target genes at 20°C, treated with 100 μg/ml FUDR and transferred to 15°C overnight and subsequently used for infection assays. Survival was quantified using standard methods (30). Animals dying of disrupted vulva or crawling on walls were censored for analysis, which was performed using Prism 9 (Graphpad Inc.). Infection assays were performed 2-3 independent times.

#### 2.2.2 Pseudomonas aeruginosa infection

Slow killing plates were made using single colony of PA14 strain was grown overnight in LB broth. 10 μl of overnight culture was spread onto slow killing assay plates and incubated at 37°C for 24 h. Plates were then placed at 25°C for a day to obtain a full bacterial lawn. Animals were grown to L4 stage on OP50 in NGM plates at 20°C. 100 μg/ml FUDR was added at L4 stage. 30-40 L4 animals of all genotypes were transferred to infection plates. 200 μg/ml FUDR was added to the infection plates. Animals were scored according to established protocols (30). Animals dying of disrupted vulva or crawling on walls were censored for analysis, which was performed using Prism 9 (Graphpad Inc.). Infection assays were performed 3 independent times.

#### 2.2.3 Enterococcus faecalis infection

A single colony of V583 strain of *E. faecalis* was grown in BHI broth at 37°C for 6-8 h. 10 μl of this culture was spread onto BHI agar plates containing 10 μg/ml kanamycin and incubated overnight at 37°C ^32^. 30-40 L4 animals grown at 20°C were then transferred onto infection plates as described (30). Animals dying of disrupted vulva or crawling on walls were censored for analysis, which was performed using Prism 9 (Graphpad Inc.). Infection assays were performed 3 independent times.

### 2.3 Longevity assays

*C. elegans* was grown at 20°C until L4 stage on NGM plates seeded with OP50 and then 100 μg/ml of FUDR was added. Animals were transferred to 25°C. Dead animals were counted every 24-48 hours. Animals that did not respond to touch with a pick were considered dead. 2-3 replicates with 20-30 animals on each plate were used for each experiment. Animals dying of disrupted vulva or crawling on walls were censored for analysis, which was performed using Prism 9 (Graphpad Inc.). Longevity assays were performed 3 independent times.

### 2.4 RT-qPCR

Animals were washed off growth or infection plates twice using sterile water and collected in 1.5 ml tubes containing 1,000 μl TRIzol (Sigma-Aldrich) for lysis. 100 μl chloroform was added and centrifuged at 12,000 rpm to separate the aqueous phase containing RNA. The aqueous phase was transferred to Purelink RNA mini kit (ThermoFisher Scientific) columns and purified total RNA was obtained following manufacturer’s instructions. cDNA was made using iScript cDNA synthesis kit (Bio-Rad). RT-qPCR was performed using SYBR Green Super mix (Bio-Rad) using a ViiA7 Real-Time qPCR system (Applied Biosystems). Test genes were normalized to neuronal reference gene *snb-1*. Fold change was calculated using the Pfaffl method (31).

### 2.5 RNA sequencing

Total RNA was collected from *C. elegans* using Purelink RNA kit as described above. Total RNA from 4 biological replicates for each condition was sent to BGI Genomics for library preparation and sequencing using BGI-seq 500. BGI provided clean reads in FASTQ format. FASTQ files were analyzed by DolphinNext high-throughput sequence analysis software (32). Steps followed in DolphinNext were as follows: FastQC was used to create quality control outputs. FASTQ files were aligned to the reference *C. elegans* genome using STAR. STAR was used to count or filter out common RNAs (eg. rRNA, miRNA, tRNA, piRNA). RSEM was used to align reads to reference transcripts and estimate gene and isoform expression levels. Genome-wide Bam analysis was done by RseQC. DEBrowser v1.22.5 was used for batch effect correction and normalization. Data analysis including generation of volcano plots and heat maps was done using DE analysis within DEBrowser v1.22.5. Adjusted p-value (P_adj_) ≤ 0.01 was considered significant for differential gene expression. Gene Ontology representation analysis was performed using the enrichment analysis tool in Wormbase (33) and g:Profiler (34).

### 2.6 Strain construction

To generate P*nhr-42::mCherry*, the *nhr-42* promoter region (2 kbp upstream of the transcription start site) was amplified and cloned into entry vector pTOPO for Gateway^®^ recombination (ThermoFisher Sci.). This amplified region was recombined into destination vector pDest-16 (generous gift by Dr. Michael Francis, UMass Chan Medical School) expressing *mCherry* to generate the expression vector. The expression vector was sequenced to verify proper insertion of the *nhr-42* promoter upstream of the mCherry coding sequence. Transformation was carried out by gonad microinjection following standard procedures (35).

### 2.7 Image acquisition

Images were taken using a Lionheart FX automatic microscope (BioTek Instruments) at 4x and 20x magnification. 30-50 C*. elegans* were used for each experimental condition. 10-20 animals at a time were anesthetized using 100 mM NaN_3_ on a 2% agar pad for imaging. All images were captured at the same exposure and intensity. Greyscale images were used for image analysis. A region of interest (ROI) was drawn around the pharynx, which showed fluorescent expression of *mCherry*. Mean fluorescence intensity (MFI) of the ROI was determined by using the Analyze>Measure function in ImageJ (NIH). MFI values were plotted in Prism 9 for statistical analysis. Fluorescence microscopy experiments were repeated 2 independent times.

### 2.8 Lipid staining

*C. elegans* was grown at 20°C until L4 stage on NGM plates seeded with OP50. 20-30 animals were placed on TSA plates containing a full lawn of *S. aureus* or BHI plates for *E. faecalis* infection. 8 h after infection, animals were washed off with water and collected in centrifuge tubes. Lipid staining using Oil Red O was performed as described (36). 10-20 animals were used for imaging. Animals were mounted on agarose pads and visualized using a Lionheart microscope at 4x and 20x magnification and the staining intensity was determined using ImageJ. Greyscale.tiff images were used for quantification. A relative threshold for the detection of red color staining was set manually (Image>Threshold>). The set threshold was constant for all images quantified. A region of interest (ROI) was drawn around each whole animal. The percent area stained inside the ROI by Oil Red O for each whole animal was calculated (Analyze>Measure>Area%). For intestine-specific quantification, the intestine (visualized by brightfield microscopy) was used as the ROI. Percent of area (Area%) stained by Oil Red O per animal was plotted and statistical analysis were performed in Prism.

### 2.9 Bacterial colony forming units

*C. elegans* was grown at 20°C until L4 stage on NGM plates seeded with OP50 or RNAi clones as mentioned. Thereafter, 30-40 animals were transferred to TSA plates containing a full lawn of *S. aureus*. At the desired timepoint 10 live animals were collected in centrifuge tubes containing autoclaved water. Colony forming units were determined as in (8). 5-7 biological replicates (10 animals per replicate) were used for each condition.

### 2.10 Quantification and statistical analysis

GraphPad Prism 9 was used for statistical analysis. Survival curves were compared using Log-Rank (Mantel-Cox test). Unpaired, two-sample two-tailed *t*-tests were performed to compare with a single reference. For multiple comparisons, one-way ANOVA was used to establish significance followed by Šidák’s *post-hoc* test. *P* ≤ 0.05 was considered significant.

## 3 Results

### 3.1 *S. aureus* induces *nhr-42* in an *hlh-30* dependent manner

**Figure 1:**
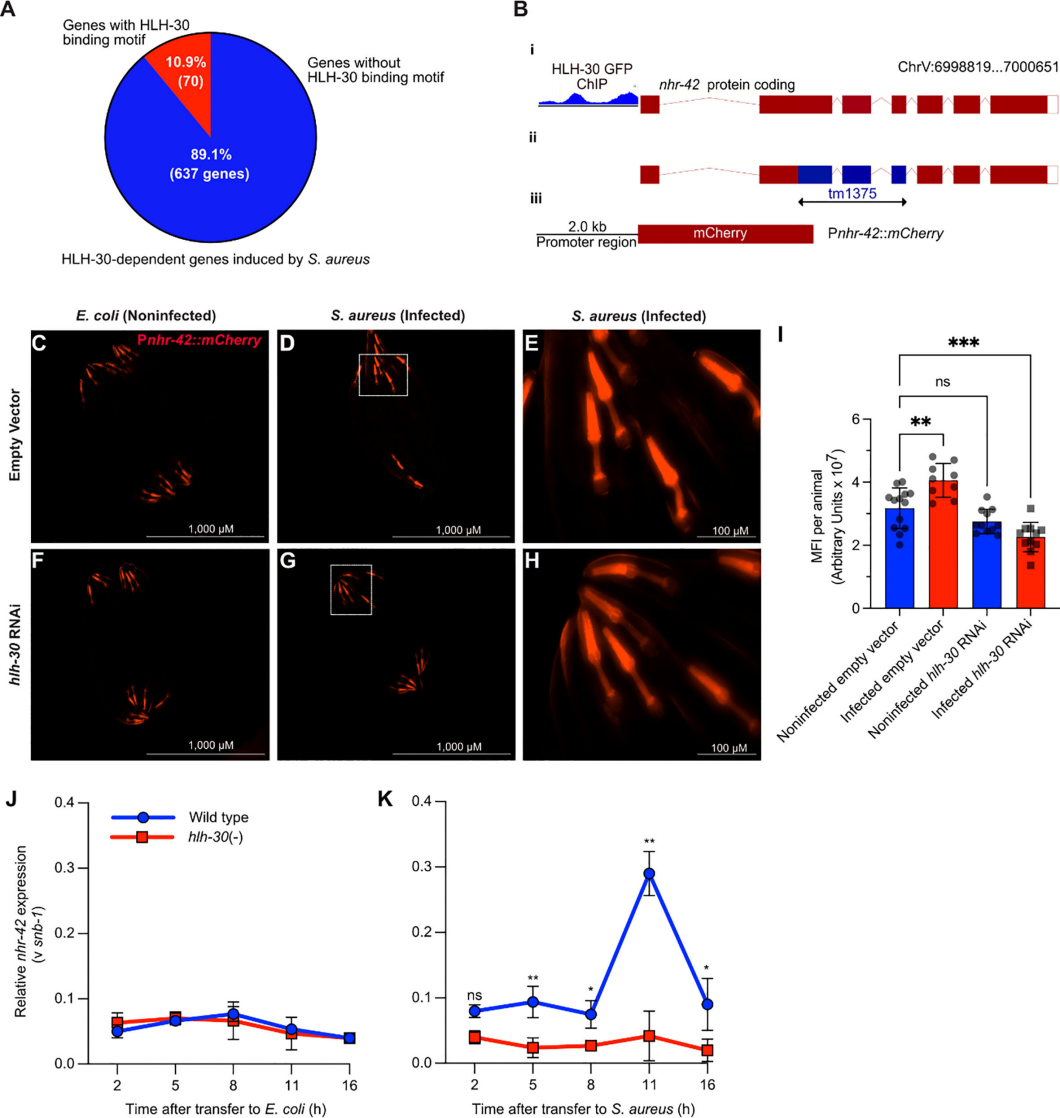
*S. aureus* induces *nhr-42* in an *hlh-30*-dependent manner **(A)** Pie chart depicting predicted genes directly induced by HLH-30 during *S. aureus* infection [Original data are from (8)]. **(B)** Cartoon depicting the *nhr-42* locus on Chromosome V, highlighting i) promoter region containing HLH-30 ChIP-seq peaks within 2 kbp of the transcription start site [original data from (37), ii) tm1375 deletion of 451 bp spanning exons 2-4,and iii) P*nhr-42::mCherry* transcriptional reporter construct from this study. **(C–H)** Epifluorescence micrographs of P*nhr-42*::*mCherry* animals fed *E. coli*, expressing RNAi empty vector or *hlh-30* dsRNA, and then exposed to *E. coli* OP50 **(C, F)** or to *S. aureus* SH1000 **(D, G)** for 24 h. E and H show magnified areas of D and G, respectively. Scale bars: 1,000 µm for images **(C, D, F, G)**, and 100 µm for E and **(H)**. Data are representative of 2 independent replicates. **(I)** Quantification of mean fluorescence intensity (MFI) per animal from **(C–H)**. n = 10 -20. **P ≤ 0.01, ****P ≤ 0.0001. ns, not significant. One-way ANOVA with Šídák’s *post-hoc* test. **(J–K)** RT-qPCR of *nhr-42* transcript in wild type and *hlh-30*(tm1978) mutants fed *E. coli* OP50 **(J)** or infected with *S. aureus* **(K)**. *P ≤ 0.05, **P ≤ 0.01. ns, not significant. 2- sided 2-sample unpaired *t*-test. 2-4 biological replicates. N = ~1,000 animals per replicate.

We previously showed that *hlh-30* induces around 600 genes during *S. aureus* infection (8). However, HLH-30 is predicted to induce just 10% of these genes by directly binding their promoters (**Figure 1A**) (8). We hypothesized that indirect regulation of key host defense genes by *hlh-30* could be mediated by the 17 transcription factors it induces during infection (8). To identify which are important in the intestinal epithelium for host defense, we performed intestine-specific silencing by RNAi. As expected, we found genes whose knockdown impaired host survival (*e.g., nhr-55*, **Figure S1A**) suggesting that they may be important for inducing the intestinal host response to infection. However, we were surprised to identify one gene (*nhr-42*), whose knockdown significantly *promoted* host survival (**Figure S1D**), suggesting that *nhr-42* may function as a host response *repressor*.

Public datasets contain evidence that HLH-30 may bind directly to the *nhr-42* promoter (**Figure 1B**) (37–39), suggesting that *nhr-42* may be a direct target of HLH-30. A fluorescent *nhr-42* transcriptional reporter showed expression mainly in the pharynx of noninfected animals (**Figure 1C**), in agreement with a previous report (40). The same reporter exhibited increased activity in the pharynx after *S. aureus* infection (**Figures 1D, E, I**), suggesting that *nhr-42* expression may be induced transcriptionally. In contrast, silencing of *hlh-30* abrogated induced reporter activity after infection (**Figures 1F–I**). These results suggested that the *nhr-42* promoter is active in the pharynx, and that its activity increases during infection, in the same tissue, in an HLH-30-dependent manner. This result is consistent with our prior RNA-seq data (8).

To gain better understanding of the timing of *nhr-42* induction, we performed time-resolved infection followed by RT-qPCR. This revealed that *nhr-42* expression is relatively stable in noninfected animals, and independent of HLH-30 (**Figure 1J**). During infection, *nhr-42* expression was elevated throughout the experiment and peaked around 11 h in wild type animals, but in *hlh-30 (*–) mutants *nhr-42* expression remained low (**Figure 1K**). These data showed that in noninfected animals *nhr-42* expression does not require HLH-30, but that its early induction and peak about 11 h during infection are HLH-30-dependent.

### 3.2 *nhr-42* represses infection survival

**Figure 2:**
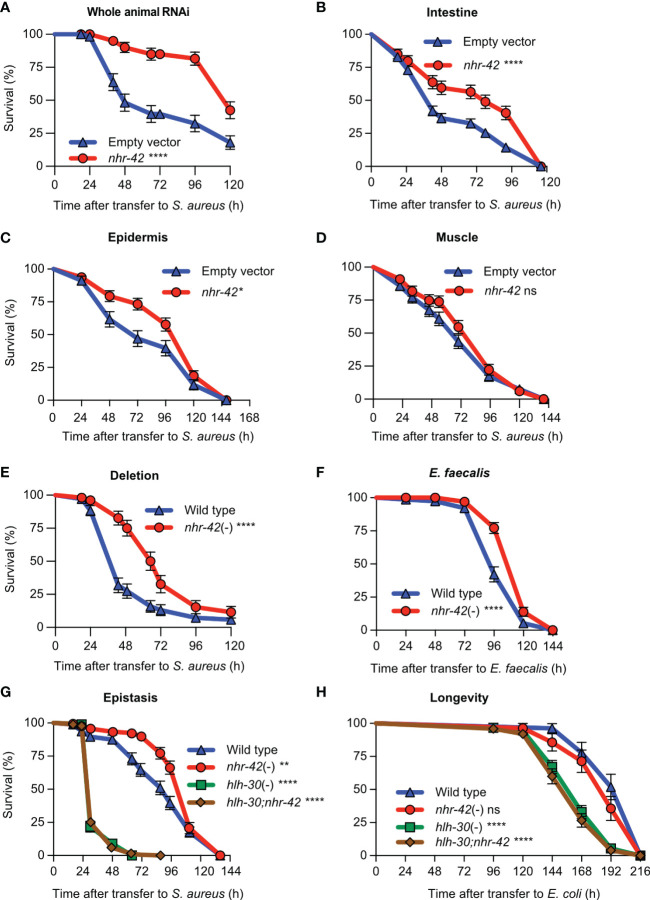
*nhr-42* represses infection survival **(A)** Survival of wild type animals fed *E. coli* HT115 expressing RNAi empty vector or *nhr-42* dsRNA during *S. aureus* SH1000 infection. Data are representative of two independent replicates. **(B)** Survival of intestine-specific MGH171 RNAi animals fed *E. coli* expressing RNAi empty vector or *nhr-42* dsRNA during *S. aureus* SH1000 infection. Data are representative of two independent replicates. **(C)** Survival of hypodermis-specific RNAi NR222 animals fed *E. coli* expressing RNAi empty vector or *nhr-42* dsRNA during *S. aureus* SH1000 infection. Data are representative of two independent replicates. **(D)** Survival of muscle-specific RNAi NR350 animals fed *E. coli* expressing RNAi empty vector or *nhr-42* dsRNA during *S. aureus* SH1000 infection. Data are representative of two independent replicates. **(E)** Survival of wild type and *nhr-42*(tm1375) animals during *S. aureus* SH1000 infection. Data are representative of 3 independent replicates. **(F)** Survival of wild type and *nhr-42*(tm1375) animals during *E. faecalis* infection. Data are representative of 3 independent replicates. **(G)** Survival of wild type, *nhr-42*(tm1375), *hlh-30*(tm1978), and *hlh-30;nhr-42* double mutants during *S. aureus* infection. Data are representative of 3 independent replicates. **(H)** Lifespan of wild type, *nhr-42*(tm1375), *hlh-30*(tm1978), and *hlh-30;nhr-42* double mutants fed *E. coli* OP50 (25°C). Data are representative of 3 independent replicates.*P ≤ 0.05, **P ≤ 0.01, ***P ≤ 0.001, ****P ≤ 0.0001. ns, not significant. Log-Rank (Kruskal-Wallis) test. N = 90-135 animals per trial.

Although we observed *nhr-42* promoter activity mainly in the pharynx, *nhr-42* could function in other tissues to repress host defense. To systematically identify the tissues in which *nhr-42* functions, we performed tissue-specific knockdown of *nhr-42* by RNAi (**Figure 2**). *nhr-42* knockdown in the whole animal strongly enhanced host survival (**Figure 2A**), providing a benchmark. As previously, intestinal knockdown enhanced survival of infection (**Figure 2B**), as also did epidermal (hypodermal) silencing (**Figure 2C**). In contrast, muscle knockdown showed no significant effect (**Figure 2D**). These data suggest that *nhr-42* represses host survival of infection mainly from the intestinal epithelium, with contributions from the epidermis.

Consistently, a total knockout of *nhr-42* provided strong protection against *S. aureus* (**Figure 2E**) and *Enterococcus faecalis*, a distantly related Gram-positive human pathogen (**Figure 2F**). However, loss of *nhr-42* did not affect defense against *Pseudomonas aeruginosa*, a Gram-negative human pathogen (**Figure S2**). These results show remarkable specificity of *nhr-42* function.

Because *nhr-42* induction by *S. aureus* is *hlh-30-*dependent, we hypothesized that they might function in the same genetic pathway. *hlh-30;nhr-42* double mutants exhibited the same susceptibility to *S. aureus* as *hlh-30* single mutants, completely suppressing the enhanced survival phenotype of *nhr-42* mutants (**Figure 2G**). Remarkably, *nhr-42* loss did not affect lifespan on nonpathogenic *E. coli*, and again *hlh-30* was epistatic to *nhr-42* (**Figure 2H**). In a linear genetic pathway model, these results would imply that *hlh-30* functions downstream or parallel to *nhr-42;* however, *hlh-30* expression was unaffected in *nhr-42* mutants (see below). Given the results so far, we rather favored a model in which *hlh-30* functions upstream of *nhr-42* to induce host defense genes that *nhr-42* represses.

### 3.3 *nhr-42* regulates genes involved in host defense and lipid metabolism

**Figure 3:**
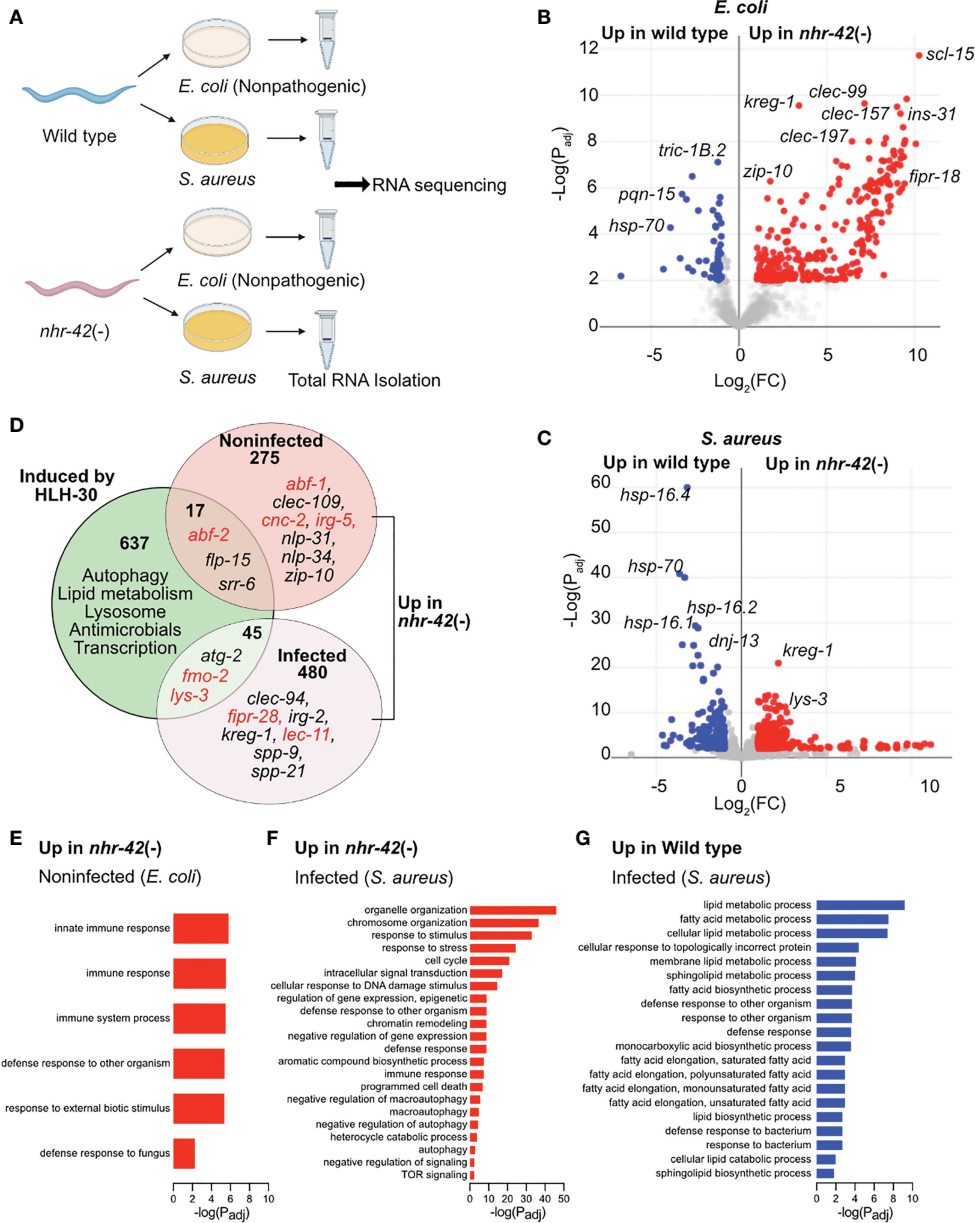
*nhr-42* regulates host defense and metabolism genes **(A)** Experimental design for RNA-seq of wild type and *nhr-42*(tm1375) animals fed *E. coli* OP50 or infected with *S. aureus* SH1000 for 5 h. **(B)** Representation of differentially expressed genes in noninfected *nhr-42*(tm1375) animals compared to wild type. Red, genes upregulated in *nhr-42*(tm1375); blue, genes upregulated in wild type. FC, fold change. P_adj_, adjusted P value. **(C)** Representation of differentially expressed genes in infected *nhr-42*(tm1375) animals compared to wild type. Red, genes upregulated in *nhr-42*(tm1375); blue, genes upregulated in wild type. **(D)** Summary of genes induced by HLH-30 during *S. aureus* infection (8) and genes that are upregulated in noninfected and infected *nhr-42*(tm1375) animals. Numbers indicate total number of differentially expressed genes in each sector. Specific examples of antimicrobial genes are provided in each sector, and relevant genes are highlighted in red. **(E)** GO over-representation among upregulated genes in noninfected *nhr-42*(tm1375) animals relative to wild type. **(F)** GO over-representation among upregulated genes in infected *nhr-42*(tm1375) animals relative to wild type. **(G)** GO over-representation among upregulated genes in infected wild type animals relative to *nhr-42*(tm1375).

To gain further insight into the function of *nhr-42*, we performed whole-animal RNA-seq of noninfected and infected animals (**Figure 3A**). We compared the transcriptomes of mutants to wild type under both conditions to identify differentially expressed genes (**Figures 3**, **S3**; **Table S1**).

292 transcripts were expressed more highly in noninfected *nhr-42* mutants compared to wild type (**Figures 3B**; **Table S1**). We found only minor overlap between this set of genes overexpressed in *nhr-42* mutants and HLH-30-induced genes in infected animals (8)(**Figure 3D**). Nonetheless, noninfected *nhr-42* mutants showed patterns that were easily identified by gene ontology (GO) over-representation analysis (**Table S2**). Specifically, relative to wild type, *nhr-42* mutants showed increased expression of genes related to various innate immunity and host defense categories (including Defense Response to Fungus) and decreased expression of genes related to the response to unfolded protein (**Figure 3E**; **Table S2**). This remarkable enrichment for host defense genes reveals that NHR-42 functions to repress host defense in noninfected animals, which is consistent with the observed enhanced survival of infection that is caused by *nhr-42* loss. Together, these results suggest that, in noninfected animals, NHR-42 represses host defense genes that are mostly HLH-30-independent.

During infection, a more complex picture emerged. *nhr-42* mutants showed increased expression of 525 transcripts (**Figure 3C**; **Table S1**), enriched for genes related to reproduction (including cell cycle and embryo development) and DNA damage response, of unclear relevance to infection biology (**Table S2**; **Figure 3F**). Perhaps easier to rationalize, they also expressed higher levels of genes related to signal transduction, response to stress, defense response (including innate immune response), apoptosis, and autophagy (including macroautophagy, negative regulation of autophagy, and TOR signaling), which are all processes that have been linked to host defense in *C. elegans* by prior research.

In contrast, infected *nhr-42* mutants showed decreased expression of 218 transcripts relative to wild type (**Table S2**). The most outstanding over-represented categories were related to lipid metabolism, including fatty acid biosynthesis and lipid catabolism (**Figure 3G**). This result prompted us to investigate the relationships among infection, lipid stores, and *nhr-42.*

### 3.4 *nhr-42* drives lipid droplet loss during infection

**Figure 4:**
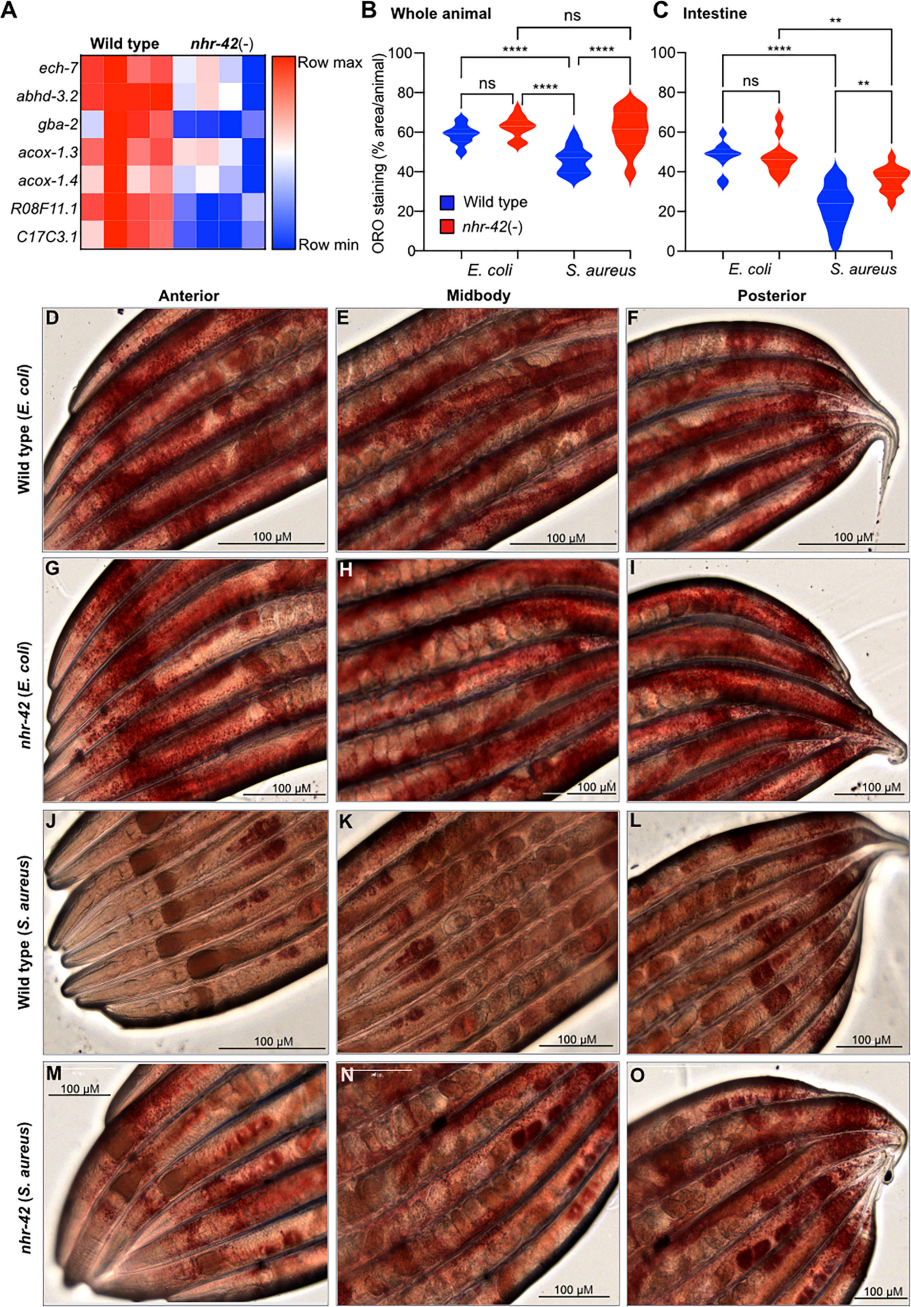
*nhr-42* is required for lipid loss during infection **(A)** Lipid catabolism genes downregulated in noninfected *nhr-42*(tm1375) mutants relative to wild type. Row-normalized FPKM values. **(B)** Quantification of Oil Red O staining in whole wild type and *nhr-42*(tm1375) animals. ****P ≤ 0.0001. ns, not significant. One-way ANOVA with Šídák’s *post-hoc* test. Representative of 3 independent replicates, 10-20 animals per condition. **(C)** Quantification of Oil Red O staining in the intestines of wild type and *nhr-42*(tm1375) animals. **P ≤ 0.01. One-way ANOVA with Šídák’s *post-hoc* test. Representative of 3 independent replicates, 10-20 animals per condition **(D–F)** ORO staining of wild type animals fed with *E. coli* OP50 for 10 h **(G–I)** ORO staining of *nhr-42*(tm1375) animals fed with *E. coli* OP50 for 10 h **(J–L)** ORO staining of wild type animals infected with *S. aureus* SH1000 for 10 h **(M–O)** ORO staining of *nhr-42*(tm1375) animals infected with *S. aureus* SH1000 for 10 h. Scale bars: 100 µm.

The RNA-seq analysis showed that *nhr-42* mutants expressed lower levels of seven known or proposed lipid catabolism genes (**Figure 4A**). These include predicted enoyl-CoA hydratase *ech-7* and acyl-CoA oxidases *acox1.3* and *acox1.4*, which are potentially involved in fatty acid β-oxidation. Also downregulated in *nhr-42* mutants was Abhydrolase domain-containing homolog *abhd-3.2*, which is predicted to be a phospholipase involved in catabolism of medium chain fatty acids. However, genes involved in lipid biosynthesis were also repressed in infected *nhr-42* mutants (**Table S2**). To determine the biological significance of these observations, we assessed the lipid status of *nhr-42* mutants by neutral lipid droplet staining with Oil Red O (ORO) (41, 42).

Noninfected *nhr-42* mutants showed a similar level of ORO staining as wild type (**Figure 4B–I**), indicating that *nhr-42* does not affect the overall levels of lipid droplets at baseline. After infection, wild type animals showed strongly decreased ORO staining (**Figures 4B, C, J–L**), whereas in *nhr-42* mutants staining was significantly preserved (**Figures 4B, C, M–O**). This indicated that *nhr-42* is required for full lipid loss during infection with *S. aureus.* Furthermore, we observed a similar defect during infection with *E. faecalis* (**Figure S4**), showing that *nhr-42* is partially required for lipid loss during infection by two archetypal Gram-positive pathogens.

### 3.5 Lipid loss and infection survival are genetically separable

**Figure 5:**
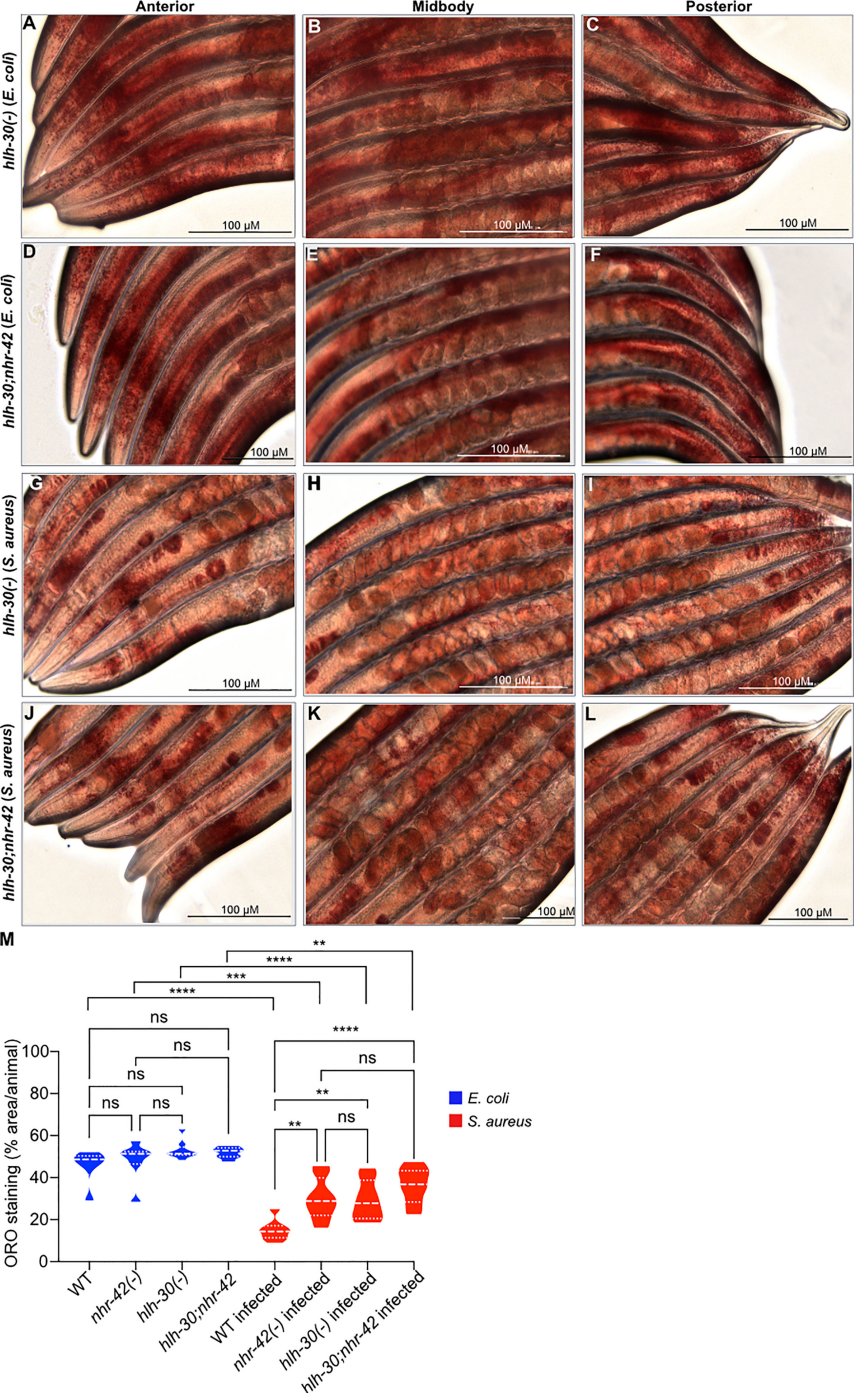
Loss of lipid droplets in *hlh-30* mutants **(A–C)** ORO staining of *hlh-30*(tm1978) animals fed with *E. coli* OP50 for 10 h. **(D–F)** ORO staining of *hlh-30*(tm1978)IV;*nhr-42*(tm1375)V animals fed with *E. coli* OP50 for 10 h. **(G–I)** ORO staining of *hlh-30*(tm1978) animals infected with *S. aureus* SH1000 for 10 h. **(J–L)** ORO staining of *hlh-30*(tm1978)IV;*nhr-42*(tm1375)V animals infected with *S. aureus* SH1000 for 10 h. **(M)** Quantification of ORO staining. 7-10 animals per condition. **P ≤ 0.01, ***P ≤ 0.001, ****P ≤ 0.0001. ns, not significant. One-way ANOVA with Šídák’s *post-hoc* test.

We and others showed that *hlh-30* is required for lipid loss during starvation (5, 6) and for host defense against *S. aureus* infection (8), suggesting that the two functions of HLH-30 may be related. Indeed, it was proposed that HLH-30 induces the expression of lipid catabolism genes that may be responsible for lipid droplet loss, at least during starvation (5). Recall that *hlh-30* was epistatic to *nhr-42* for infection survival (**Figure 2G**) *i.e.*, *hlh-30;nhr-42* double mutants are defective in infection survival, like the *hlh-30* single mutants. To determine the relationship between this phenotype and lipid loss, we performed ORO staining of the *hlh-30;nhr-42* double mutants (**Figure 5**). *hlh-30;nhr-42* double mutants were not significantly different from wild type, *hlh-30*, or *nhr-42* animals at baseline (**Figures 5A–F, M** **)**. After infection with *S. aureus*, the *hlh-30* single mutants showed a much smaller drop in ORO staining compared to wild type, to a level similar to the *nhr-42* single mutants (**Figures 5G–I, M** **)**. Moreover, the *hlh-30;nhr-42* double mutants were statistically indistinguishable from the single mutants (**Figures 5J–M**). Therefore, *hlh-30* is also required for lipid loss during *S. aureus* infection, and by epistasis participates in the same genetic pathway as *nhr-42.* However, loss of *hlh-30* impairs host infection survival while loss of *nhr-42* promotes it; therefore, these mutations have opposite effects on infection survival but the same effect on lipid loss. These data support the notion that *nhr-42* mediates the lipid loss function of *hlh-30*, and thus that HLH-30 does not directly drive lipid loss during infection. In further support of this notion, of the lipase-like (lipl) genes that were proposed to function downstream of HLH-30, only *lipl-2* was differentially expressed by RNA-seq, and only in one condition: infected *nhr-42* mutants expressed more *lipl-2* compared with infected wild type (**Table S1**). Therefore, the defective lipid loss of *nhr-42* mutants is likely not due to decreased lipl gene expression. It follows that infection survival and lipid loss have distinct genetic requirements, and that lipid droplet loss does not explain the enhanced resistance to infection of *nhr-42* mutants.

### 3.6 *nhr-42* represses host defense genes required for enhanced survival during infection

**Figure 6:**
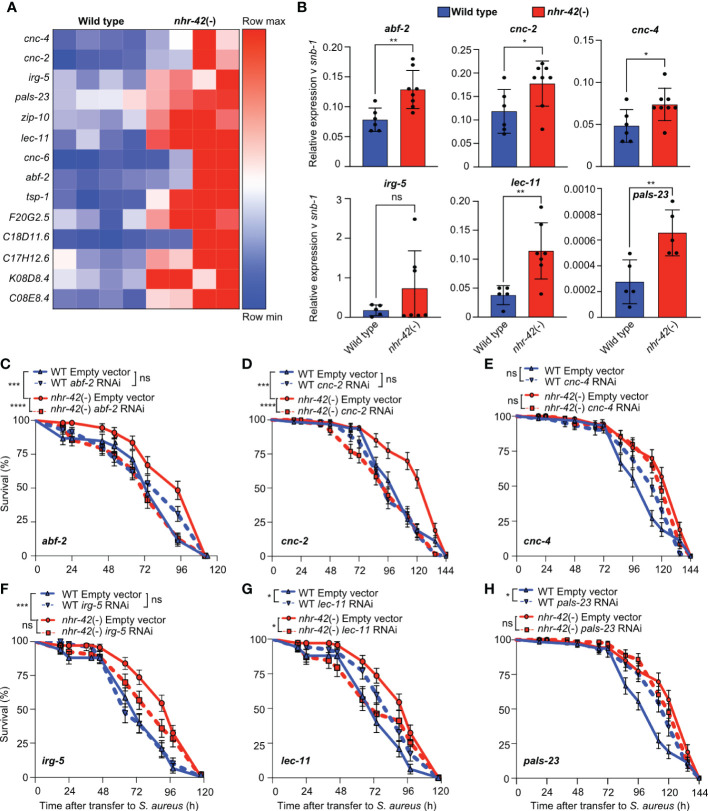
Antimicrobial genes required for enhanced infection survival of *nhr-42* mutants **(A)** Host defense genes upregulated in noninfected *nhr-42*(tm1375) mutants relative to wild type. Row-normalized FPKM values. **(B)**RT-qPCR of transcripts for select antimicrobial genes in noninfected wild type and *nhr-42*(tm1375) mutants. 5-8 biological replicates. N = ~1,000 animals per replicate. *P ≤ 0.05, **P ≤ 0.01. ns, not significant. *t*-test. **(C)** Infection survival of wild type and *nhr-42*(tm1375) animals fed *E. coli* HT115 expressing RNAi empty vector or *abf-2* dsRNA DNA prior to infection with *S. aureus* SH1000. Data are representative of 3 independent replicates. **(D)** Infection survival of wild type and *nhr-42*(tm1375) animals fed *E. coli* HT115 expressing RNAi empty vector or *cnc-2* dsRNA prior to infection with *S. aureus* SH1000. Data are representative of 2 independent replicates. **(E)** Infection survival of wild type and *nhr-42*(tm1375) animals fed *E. coli* HT115 expressing RNAi empty vector or *irg-5* dsRNA prior to infection with *S. aureus* SH1000. Data are representative of 2 independent replicates. **(F)** Infection survival of wild type and *nhr-42*(tm1375) animals fed *E. coli* HT115 expressing RNAi empty vector or *pals-23* dsRNA prior to infection with *S. aureus* SH1000. Data are representative of 2 independent replicates. **(G)** Infection survival of wild type and *nhr-42*(tm1375) animals fed *E. coli* HT115 expressing RNAi empty vector or *cnc-4* dsRNA prior to infection with *S. aureus* SH1000. Data are representative of 2 independent replicates. **(H)** Infection survival of wild type and *nhr-42*(tm1375) animals fed *E. coli* HT115 expressing RNAi empty vector or *lec-11* dsRNA prior to infection with *S. aureus* SH1000. Data are representative of 2 independent replicates. *P ≤ 0.05, ***P ≤ 0.001, ****P ≤ 0.0001. ns, not significant. Log-Rank (Kruskal-Wallis test). The Empty Vector controls for **(D, E, H)** are the same. The Empty Vector controls for F and G are the same. In **(C–H)** N = 90-135 animals per trial.

As mentioned, noninfected *nhr-42* mutants displayed a strong signature of upregulated host defense genes (**Figure 3E**). Such upregulated genes include antibacterial factor-related genes *abf-1* and *abf-2*, antimicrobial caenacins *cnc-2, cnc-4, and cnc-6*, and immune response gene *irg-5*, as well as *lec-11* and *pals-23*, which were previously implicated in host defense (**Figure 6A**) (43–50). RT-qPCR independently confirmed their upregulation, except for *irg-5* (**Figure 6B**). To test the hypothesis that antimicrobial gene upregulation may be a major contributor to the enhanced infection survival of *nhr-42* mutants, we performed RNAi-mediated knockdown. Silencing of *cnc-4, irg-5*, and *pals-23* had no significant effect in *nhr-42* mutants, whereas silencing of *abf-2, cnc-2*, and *lec-11* significantly decreased their enhanced survival (**Figures 6C–H**). *abf-2* and *cnc-2* knockdowns showed the strongest effects in *nhr-42* mutants, but did not affect the wild type (**Figures 6C, D**). Curiously, silencing of *lec-11* (which reduced the survival of *nhr-42*) and of *pals-23* (which did not), both enhanced the survival of wild type (**Figures 6G, H**). Thus, specific antimicrobial effectors that are constitutively induced in *nhr-42* mutants are required for their enhanced infection survival.

### 3.7 *abf-2* mediates infection resistance in *nhr-42* mutants

**Figure 7:**
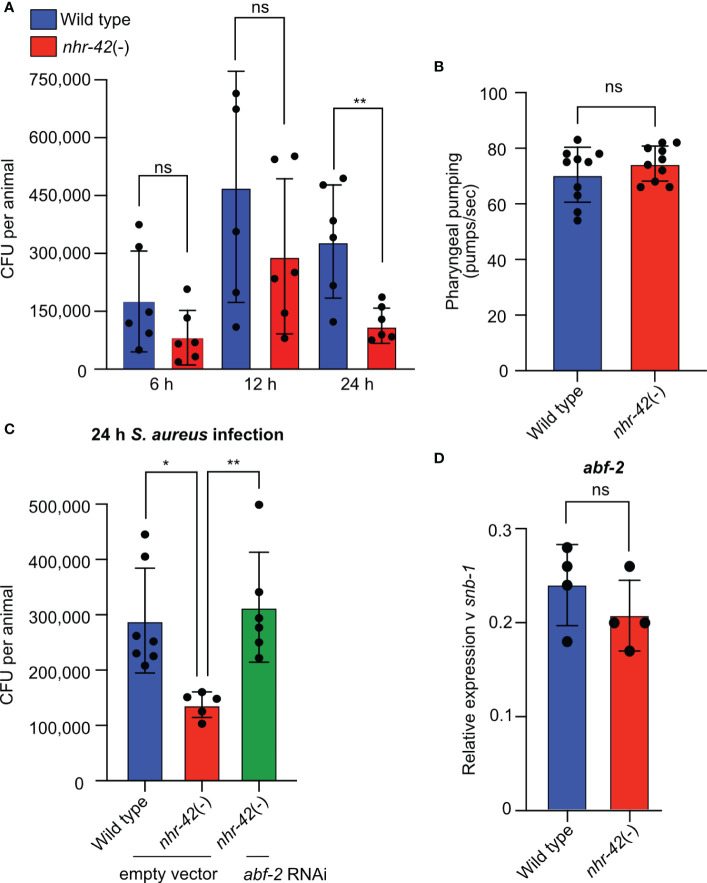
*abf-2* mediates infection resistance in *nhr-42* mutants **(A)** *S. aureus* SH1000 colony forming units (CFU) from wild type and *nhr-42*(tm1375) animals after 6, 12, and 24 h of infection. Each dot represents a single animal (N = 6 per time point). Means ± SD. **(B)** Pumping rates of noninfected wild type and *nhr-42*(tm1375) animals. Each dot represents a single animal (N = 10). Means ± SD. **(C)** *S. aureus* SH1000 CFU from wild type and *nhr-42*(tm1375) animals fed *E. coli* HT115 expressing RNAi empty vector or *abf-2* dsRNA prior to infection for 24 h. Each dot represents a single animal (N = 5-7). Means ± SD. **(D)** RT-qPCR of *abf-2* transcript in infected wild type and *nhr-42*(tm1375) animals. Each dot represents a single biological replicate of ~1,000 animals. Means ± SD. * *P*≤ 0.05, ** *P*≤ 0.01. ns, not significant. 2-sided 2-sample unpaired *t*-test.

To determine if *nhr-42* mutants show enhanced infection survival because of enhanced infection resilience (*i.e.*, same colonization as wild type) or resistance (*i.e.*, lower colonization than wild type), we measured *S. aureus* accumulation in the *C. elegans* gut over time. Whereas by 24 h of infection the *nhr-42* mutants accumulated significantly less *S. aureus* than wild type (**Figure 7A**), they tended to accumulate less at earlier time points as well. Control experiments showed that the pharynxes of *nhr-42* mutants pumped at rates that are indistinguishable from wild type (**Figure 7B**), suggesting that the decreased accumulation of *S. aureus* in *nhr-42* mutants was not due to decreased oral intake. Of the genes that were required for enhanced survival of *nhr-42* mutants, *abf-2* is the only one whose product has demonstrated antibacterial activity *in vitro* (44), and therefore we chose it for further study. Animals treated with *abf-2* RNAi showed increased *S. aureus* load, comparable to wild type (**Figure 7C**), suggesting that *abf-2* mediates infection resistance in *nhr-42* mutants. As mentioned, noninfected *nhr-42* mutants exhibit higher expression of *abf-2* than wild type counterparts (**Figures 6A, B**). However, *abf-2* expression at 5 h infection was similar in wild type and *nhr-42* mutant animals (**Figure 7D**), suggesting that *abf-2* may be overexpressed only in noninfected *nhr-42* mutants. However, these data do not rule out potentially increased *abf-2* expression in *nhr-42* mutants at other times of infection.

## 4 Discussion

We describe studies that implicate the HLH-30-dependent transcription factor gene *nhr-42* as an important modulator of host defense and of lipid droplet metabolism. Although the exact molecular and organismal relationships between these two processes requires further study, our data demonstrate that downstream regulatory target genes that are induced by HLH-30 play central roles in infection survival and organismal lipid metabolism.

*nhr-42* is a previously uncharacterized member of the nuclear receptor family, which is amplified in *C. elegans* (22). For this reason and because of evolutionary drift, establishing clear one-to-one orthology with human genes is challenging. Nonetheless, the presence of a coiled-coil domain N-terminal to the ligand binding domain and homology-based predictions by Ensembl Compara suggest that NHR-42 may be related to mammalian NR1D1 (also known as REV-ERBα). In mammalian innate immune cells, NR1D1 represses TLR4 and pro-inflammatory genes, such as CCL-2 and IL-6 (51–53). Pharmacological NR1D1 activation represses pro-inflammatory cytokine secretion and promotes IL-10 in primary rat microglia (54), in murine models of colitis (55), in the lungs of murine models of inhaled LPS (56), in the livers of murine models of fulminant hepatitis, and in murine and human macrophages (57). In murine and human gastric epithelial cells, NR1D1 represses antimicrobial peptides and CCL-21, repressing host defense and promoting *Helicobacter pylori* infection (58). *Nr1d1^-/-^* mice show spontaneous microglial activation and enhanced hippocampal neuroinflammatory responses to systemic LPS (59). Moreover, recent work showed that murine TFEB/TFE3 induce *Nr1d1* expression during fasting in the liver (60) and that NR1D1 induces the expression of *TFEB* in human macrophage-like THP-1 cells (61). Thus, although published precedent supports NHR-42 as a functional homolog of NR1D1, the regulation of NHR-42/NR1D1 by HLH-30/TFEB during infection is a novel and important interaction that could be exploited for therapeutic purposes. As a nuclear receptor, NHR-42 is expected to bind to lipophilic ligands, but their identity and biological functions remain unknown. Moreover, promoter reporters show that *nhr-42* and its repression target *abf-2* are expressed in the pharynx. It is unclear why HLH-30, which is expressed throughout the soma (8), induces *nhr-42* only in this tissue. One possibility is that a second necessary factor, possibly a transcriptional co-regulator, is pharynx-restricted.

ABF-2 is a 62-residue peptide homologous to *Ascaris suum* antibacterial factor (ASABF) and carries a conserved arthropod defensin consensus motif (44). It is one of a remarkably small number of *C. elegans* antimicrobial effectors with experimentally demonstrated bactericidal activity. *In vitro*, recombinant ABF-2 exhibits antimicrobial activity against Gram-positive and -negative bacteria, as well as yeasts (44). The expression of gene *abf-2*, which curiously resides together with paralog *abf-1* within an intron belonging to uncharacterized gene *C50F2.2*, is induced by *Salmonella-*infected animals in a manner that requires the sole *C. elegans* toll-like receptor gene, *tol-1* (62, 63). Additionally, *abf-2* is upregulated in long-lived *age-1* and immune-deficient *dbl-1* mutants, in a manner that requires *daf-16* and *nsy-1* (of the insulin signaling and p38 MAPK pathways, respectively) (64). Additionally, *abf-2* is induced by *S. aureus* and is HLH-30-dependent (8), which illustrates how NHR-42 can repress antimicrobial genes that are HLH-30 targets. Remarkably, loss of *nhr-42* did not protect animals from *P. aeruginosa.* This may reflect how remarkably different the host responses to *S. aureus* and *P aeruginosa* are (10). Alternatively, loss of *nhr-42* may better counteract the pathologies caused by *S. aureus*, which are markedly different those caused by *P. aeruginosa* (10). It is worth noting that ABF-2 has not been shown to exert antibacterial activity against *P. aeruginosa* (44). Nevertheless, our results highlight how constitutively increased expression of *abf-2* may protect *C. elegans* from *S. aureus* in *nhr-42* mutants. To our knowledge, this is the first report of the *in vivo* importance of ABF-2 in host infection resistance.

In addition to its host defense gene repressive role, we discovered that *nhr-42* promotes the loss of lipid stores during infection. Previously, HLH-30 was proposed to induce lipid mobilization by direct induction of lipase genes (5). However, our results suggest that this mechanism may be secondary to another, which is *nhr-42-*dependent. What this *nhr-42-*dependent mechanism might be is speculation. Whether lipids are lost in wild type animals due to increased consumption (mobilization) or decreased biosynthesis remains an open question. Silencing of lipid catabolic genes that are downregulated in *nhr-42* mutants did not prevent lipid droplet loss in infected wild type animals (not shown), thereby not supporting the hypothesis that downregulation of these genes individually does not account for decreased lipid droplets in *nhr-42* mutants. Moreover, RNA-seq showed that several genes related to lipid biosynthesis were downregulated in infected *nhr-42* mutants compared to wild type, which would be expected to deplete lipid droplets rather than preserve them.

Equally enigmatic is the role that lipid droplet depletion plays in host defense. Although lipid droplet depletion has been observed during infection with other model pathogens (65, 66), whether it aids or hinders host defense is unknown. Additionally, mutants in which the stress-response transcription factor SKN-1/NRF2 is hyperactivated exhibit lipid depletion and enhanced infection survival (66). However, the mechanistic relationship between the two correlated phenomena is currently unknown. Lipid species can perform a broad range of biological functions, including energy storage, building blocks for biosynthesis of metabolites and organelles, membrane and cellular repair, antibacterial activity, and signaling as regulatory ligands for enzymes and transcriptional regulators. Our previous data show that *S. aureus* causes wholesale disruption of the apical domain of the intestinal epithelium, followed by cytolysis of the epithelium and underlying tissues (10). We also have found that autophagy, which requires membrane rearrangements and is involved in membrane repair and damaged organelle recycling, is required for defense against *S. aureus* (8). Thus, existing evidence supports a role of lipids in host cytoprotection. However, other roles (energy, signaling, bactericide) cannot be ruled out and deserve further exploration. Lipid stores serve as sources of energy and as building blocks for oogenesis (67). In absence of lipid mobilization, *nhr-42* mutants are expected to obtain energy for host defense from alternative sources: one possibility could be autophagy-mediated degradation of macromolecules and organelles. Remarkably, reduced lipid mobilization in *nhr-42* mutants did affect their brood sizes (not shown). Therefore, *nhr-42* mutants may be adapted to obtain energy for biosynthesis and cytoprotection from sources other than lipid droplets, and their enhanced infection resistance does not appear to be related to reproduction.

There remain many plausible answers to what purpose induction of *nhr-42* by HLH-30 may serve during infection. If we assume that NHR-42 mainly functions as a direct repressor of gene expression, its induction may help terminate the host response after infection clearance. This is not allowed to happen by design in our infection assays to facilitate experimental study, but in the wild animals are free to deploy behavioral host defense and thus physically avoid ingesting pathogenic bacteria. The hypothetical direct repressor activity of NHR-42 may (or may not) be regulated by yet unidentified lipophilic ligands, as is the case for better understood nuclear receptors. Alternatively, NHR-42 may function indirectly, by inducing the expression of an unknown and intermediate repressor of gene expression. In either scenario, the net effects of NHR-42 activity would be to suppress host defense. Future work must examine these mechanistic scenarios and their physiological significance.

## Data availability statement

The RNA-seq data are deposited in the Sequence Read Archive of the National Library of Medicine of the USA, accession number PRJNA929270 (https://www.ncbi.nlm.nih.gov/bioproject/PRJNA929270/).

## Author contributions

Experimental conception and design: JI, DG, SL. Data analysis: DG, XG, SL, JI. Experimentation: DG, XG, SL. Writing and editing: DG, XG, SL, JI. All authors contributed to the article and approved the submitted version.
