# Supplementary material for: C. elegans orphan nuclear receptor NHR-42 represses innate immunity and promotes lipid loss downstream of HLH-30/TFEB
Source: Front Immunol. 2023 Feb 13;14:1094145. doi: 10.3389/fimmu.2023.1094145 (PMC9968933; doi:10.3389/fimmu.2023.1094145)
Supplement: Supplementary file 1 [file DataSheet_1.docx]

Supplementary Material

## Figure S1. Intestinal RNAi of HLH-30-dependent transcription factors

MGH171 animals were fed *E. coli* expressing RNAi empty vector or *nhr-55* (**A**)*, nhr-18* (**B**)*, nhr-21* (**C**)*, nhr-42* (**D**)*, klf-1* (**E**)*, nhr-134* (**F**)*, nhr-193* (**G**)*, nhr-180* (**H**)*, and nhr-105* (**I**) dsRNA prior to *S. aureus* SH1000 infection. ** *P* ≤ 0.01, **** *P* ≤ 0.0001. Absent symbols represent ns, not significant. Log-Rank.

**Figure S2. *nhr-42* does not affect survival of *P. aeruginosa* infection**

Survival of wild type and *nhr-42*(tm1375) of *P. aeruginosa* PA14 infection. ns, not significant (Log-Rank, Kruskal-Wallis test).

**Figure S3. Differential gene expression analysis of *nhr-42* mutants and wild type**

Heat map representation of gene clusters that are differentially expressed between wild type and *nhr-42*(tm1375) animals, fed *E. coli* OP50 (**A**) or *S. aureus* SH1000 (**B**) for 5 h. Columns are 4 biological replicates, rows are genes. Colors encode log_2_(FC).

**Figure S4. *nhr-42* promotes loss of lipid droplets during *E. faecalis* infection.**

1. ORO staining of wild type animals fed with *E. coli* OP50 for 10 h.
2. ORO staining of wild type animals infected with *S. aureus* SH1000 for 10 h.
3. ORO staining of *nhr-42*(tm1375) animals fed with *E. coli* OP50 for 10 h.
4. ORO staining of *nhr-42*(tm1375) animals infected with *S. aureus* SH1000 for 10 h.
5. Quantification of ORO staining. 7-10 animals per condition. ** *P*  ≤ 0.01, **** *P* ≤ 0.0001. One-way ANOVA.

**Supplementary Table 1. Differential gene expression analysis of wild type and *nhr-42*(tm1375) animals.**

**Supplementary Table 2. Gene ontology analysis of differentially expressed genes in wild type relative to *nhr-42*(tm1375) animals.**
